# Rehabilitation therapy for children with autism based on interactive VR-motion serious game intervention: a randomized-controlled trial

**DOI:** 10.3389/fpubh.2025.1628741

**Published:** 2025-09-04

**Authors:** Xufeng Ma, Kai Song

**Affiliations:** ^1^College of Arts and Media, Tongji University, Shanghai, China; ^2^School of Arts, Beijing Language and Culture University, Beijing, China

**Keywords:** autism spectrum disorder, serious games, virtual reality, game therapy, social communication

## Abstract

**Background:**

Autism Spectrum Disorder (ASD) poses complex challenges in social communication, behavior, and learning, which traditional therapies often fail to fully address. This study explores an interactive VR-Motion serious game designed for children with ASD, leveraging immersive, controlled environments to enhance social skills, self-care, and emotional regulation.

**Objective:**

Children with autism often suffer from multiple complications; thus, new serious games that are versatile and easy to use are more suitable for them. This study aims to design and develop a versatile serious game for multifaceted intervention in children with autism, encompassing psychological, social, and learning aspects. We also aim to evaluate the rationality of its design and the effectiveness of its content, with the ultimate goal of providing an effective intervention therapy for children with autism.

**Subjects and methods:**

This study used the methods of pre-experiment and formal experiment. The pre-experiment (*n* = 2): Control group (Average Age = 123.8 months, SD = 32.27 months) experimental group (Average Age = 119.4 months, SD = 29.41 months), in which one male and one female was trained 4 times a week for 4 h each time for 3 weeks. The formal experiment (*n* = 19): random number sampling method was adopted to sample them into two groups for simultaneous intervention training, 4 times a week for 4 h each time for 16 weeks. (Average Age: 98.76–150.23 month, SD: 17.79–44.81 month, $G_{\mathrm{Sex}\;\text{ratio}}$ : 5:4).

**Results:**

The analysis of formal experimental data shows that the design of this interactive VR-Motion serious game is reasonable and it has a good effect on the training of social communication and self-living ability of autistic children. In the post-test of wish tracking, df = 9; *t* = −1.155 and *p* = 0.281, it can be seen that the interactive VR-Motion serious game intervention has a good improvement on the social willingness of autistic children.

**Conclusion:**

The experimental results show that the design of interactive VR-Motion serious game provides excellent guidance for the simulation of autistic children’s attempts to socialize and experience warm social emotions and introduces correct life situations by cultivating their independent willingness to join social interactions during the game, and it has good benefits in establishing a stable learning environment.

## 1 Introduction

Game therapy is an effective approach in rehabilitating autistic children. It utilizes games to enhance understanding of the patients’ psychology, their perception of daily life and their ability to connect with others. Capitalizing on the inherent characteristics of serious games, game therapy seeks to engage the participants’ intrinsic motivations, fostering self-healing and aiding in their transition back to a normative lifestyle. In intervention methods, symbolic play, language skills, joint attention and social interaction intervention approaches are widely applied and acknowledged (1). As a customized activity, games provide a comfortable medium for expressing emotions and revealing true thoughts through action.

In addition, as a comprehensive system, virtual reality integrates multimedia, multi-perception and other disciplines, which is a computer simulation system that can create and experience the virtual world. It has four characteristics including multi-perceptibility, sense of presence, interactivity, autonomy and has unique advantages for interventions in autistic children. Li et al. (2) conducted a systematic review and meta-analysis on VR interventions for Autism Spectrum Disorder (ASD), emphasizing the need for more rigorous research designs to validate the effectiveness of VR in social and affective skill training. Zhao et al. (3) found that integrating VR with traditional rehabilitation significantly enhanced cognitive and social communication skills in children with ASD, demonstrating VR’s potential to augment conventional therapeutic approaches. Frolli et al. (4) reported that VR interventions could effectively reduce the time required for children with ASD to learn to recognize and use primary and secondary emotions, suggesting that VR is a promising tool for emotional training. Dan (5) reviewed the challenges individuals with ASD face in daily tasks and social interactions, proposing VR as a trans-formative solution that could significantly improve social skills and daily autonomy. Some studies have utilized VR-related technologies (6), with immersive interactive experiences being considered the key factor in producing effective outcomes (7). These innovative methods can not only be applied in the clinical field as tools to assist therapists (8) but also in the educational domain to help ASD children develop their communication and social skills (9). This narrative review suggests serious games, particularly in virtual settings, may offer promising and controlled tools for social skill development in autistic children (10).

Serious games have been gradually used in the rehabilitation training of autistic children for the following reasons: Firstly, autistic children focus on visual learning and immersive somatic interactive games with the computer interface to present teaching content are more in line with their cognitive characteristics (11). Secondly, immersive somatic interactive games can provide controllable teaching contents, which can reduce the requirement for interpersonal interaction, reduce the anxiety level in children with autism and create a relatively safe learning environment (12). Sha et al. (13) created a VR game through unity and selected three autistic children for testing. The results showed that children’s attention was improved to varying degrees compared to traditional rehabilitation training and all of them showed a strong interest in games. The study presents a participatory design framework and demonstrates its potential to improve attention and emotion recognition through the serious game SALY. Neuroscience & Biobehavioral Reviews (10). The review reveals that serious games significantly improve social skills in autistic individuals, as supported by most studies examined (14).

According to the survey, we found that Art-Therapy have also been recognized by international research, as the “ninth art” of the game, its essence is creative, interactive and expressive media, more vivid and attractive than language and contribute to the formation of multi-modal interaction (15). This notion is particularly evident in game-based interventions for ASD (16). It is also why symbolic interactive games can be a typical approach to rehabilitation interventions for ASD (17). Psychomotor interventions using serious games and assistive robots significantly improved postural balance and motor coordination in children with autism (18). Therefore, we carry out technical research and program design of a VR sports education simulation and provide an interactive 3D dynamic live action and physical behavior system simulation by using the powerful simulation environment of VR technology to integrate multi-source information, so that autistic children can easily immerse themselves in an animal education simulation environment, thus creating a virtual environment suitable for the emotional sustenance of autistic children. It helps autistic children overcome social phobia, willing to communicate and open themselves for better rehabilitation and training.

## 2 Subjects and methods

### 2.1 Participants and sample-size justification

An *a priori* power analysis was performed with G*Power 3.1 (two-tailed, independent-samples *t*-test; allocation ratio 1:1). To buffer a projected 10% attrition, we aimed for 24 enrolments (12 per group). Logistical constraints—including academic-calendar limits and restricted headset inventory—ultimately confined recruitment to 20 participants (10 per arm). We mitigated this limitation by: (i) reporting all outcomes with 95% confidence intervals and standardized effect sizes; (ii) supplementing primary tests with Bayesian estimation to quantify evidence strength; and (iii) highlighting power constraints in the discussion, positioning this single-centre trial as a hypothesis-generating precursor to an upcoming multi-site study.

### 2.2 Game description

**Figure 1:**
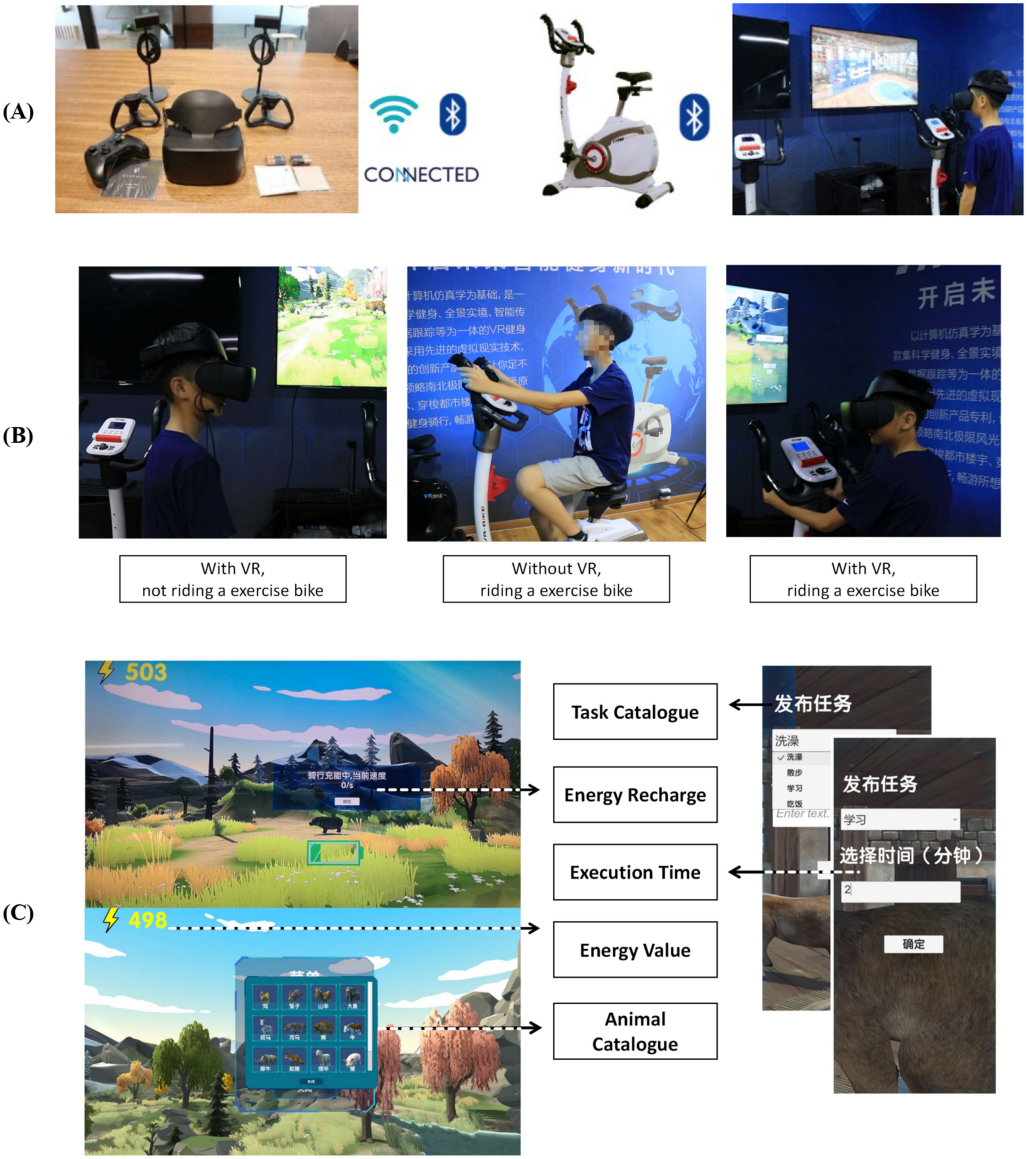
Game components **(A)**, Game control methods **(B)**, Game operation interface **(C)**.

In the game, children can choose their favorite animals to raise, give them names, and assign tasks such as taking a bath, going for a walk, having a meal, or going to sleep. At the bottom of the interface, they can enter a numerical value to specify the length of the activity. During this process, the animal will communicate with children in real time, provide feedback, and create a cordial atmosphere, thus aiding in the therapy for autism. The animals will make friends during their walks in the wild and they will bring their friends to their homes. This design encourages autistic children to open up, overcome their fears, make friends, and improve their social skills. Energy is consumed when children assign tasks. When the energy level is low, children need to pedal a stationary bicycle to replenish energy in the real world, as shown in Figure 1. This is a perfect combination of cycling sports and games and it is scientifically proven that exercise makes children happy and is more beneficial to the rehabilitation of autistic children (19).

### 2.3 Game experimental setup and procedure

The game can be operated with or without VR headsets and is compatible with computers, smartphones, and other mobile devices. The procedure is as follows:

1. VR Headset Use: Children wear VR headsets, while the teacher releases tasks on the computer. After communicating with the children and obtaining their consent, the teacher clicks “OK,” and the animal walks to the task target point. The clock starts once the animal reaches the target point.
2. Control Mechanism: The child controls the character’s movement primarily through the exercise bike. Pedaling the bike generates energy, which is used to power various in-game activities. This ensures that the child’s physical activity is directly linked to their progress in the game, creating a feedback loop that encourages both physical exercise and engagement with the game.
3. Indoor and Outdoor Activities: The animal’s activity area is divided into indoor and outdoor environments. When the animal exits the indoor space, the scenario shifts to a forest setting, which the child can explore using the VR headset. During this time, the teacher can help the child recognize different animals through an in-game menu. The teacher can also end the task or return the animal to its indoor space through the menu, facilitating learning and interaction for the child.
4. Teacher Assistance: The game design includes assistance from a third-party teacher to increase communication between the teacher and the autistic child during gameplay. The immersive virtual game uses sound, pictures, videos, and animations to stimulate the child’s interest, promote participation, and alleviate fears. During sessions teachers: (a) deliver pre-task social stories; (b) issue “guided choice” prompts when the child stalls > 15 s; (c) model appropriate language using aided AAC boards; and (d) score session fidelity on a 10-item checklist. Weekly supervision ensures protocol adherence. This structured mediation aligns with peer−/teacher-assisted models shown to enhance generalization of social skills.

### 2.4 Technical requirements and deployment scenarios

Peripheral Devices: A cadence-sensor bicycle with ANT+/Bluetooth transceiver (US $120) supplies real-time power output; any alternative stationary bike can be integrated via a serial-COM bridge. For motion tracking without head-mounted display (HMD) the system recognises keyboard, gamepad, or touch input, ensuring accessibility when VR optics are unaffordable.

Software Stack: Unity 2021.3 LTS, OpenXR runtime, and SQLite local database; Python 3.10 scripts perform data logging and anonymised batch export. The build operates fully offline and synchronises via Wi-Fi 802.11n when connectivity permits, satisfying General Data Protection Regulation (GDPR) and local privacy statutes.

Adaptations for Low-Resource Settings: Opt-in “2D-lite” mode disables high-poly assets and substitutes prerecorded 720p video loops, reducing GPU load ≈ 60%.

Text strings and voice-overs are externalized in JSON, enabling rapid localization. All source code and 3-D assets (except licensed music) are released under CC-BY-NC 4.0 on GitHub; hardware schematics for the bike-interface PCB are included to encourage local fabrication.

These specifications align with recent VR-CORE recommendations for clinical trials in immersive environments, facilitating replication and scalability across diverse socioeconomic contexts.

### 2.5 Game design requirements

In the realm of game design, the conceptualization of systemic design strategies is crucial to the game’s efficacy. Based on Raaja Shri (20) research theory and practical experience, combined with our actual research, we have concluded that there are three main aspects of game design. First, provide information presentation that fits the autistic children’s personality thinking, which can emphasize the system’s ability to create visual and auditory interventions for the patient. Second, to create a safe intervention environment for autistic children, compared with normal children, autistic children have speech and behavior disorders. Virtual reality games should consider the behavioral characteristics of patients and create a safe and efficient intervention environment. Third, provide personalized treatment for autistic children, simple game content models were summarized that could provide a wide range of treatments for children with autism. The intervention is deliberately designed so that children with autism need neither prior technical literacy to interact with the game nor any previous gaming experience during the experimental sessions.

**Figure 2:**
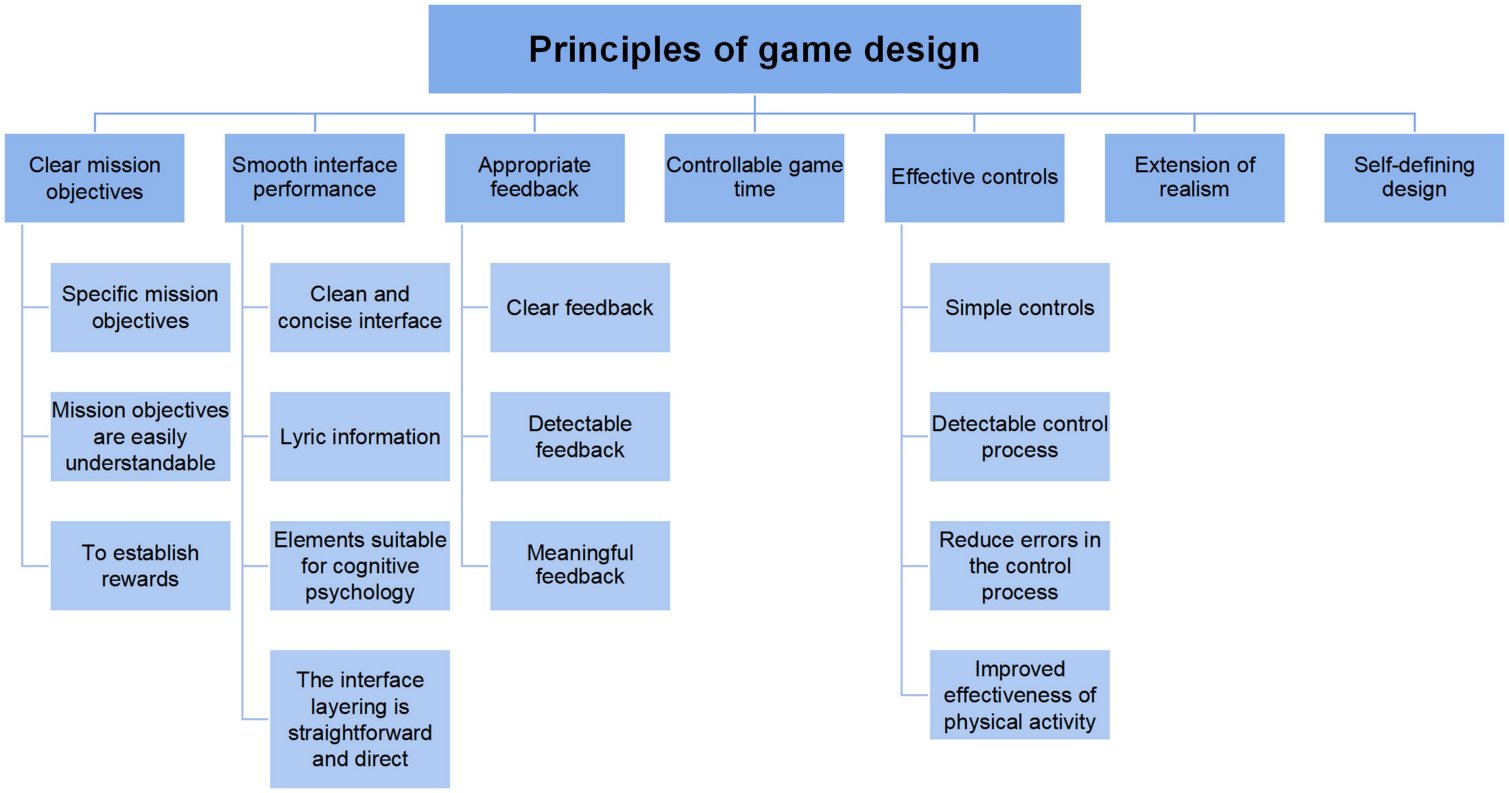
Serious game design principles for the intervention of children with Autism in rehabilitation therapy.

From the above three aspects lead to the following principles for designing serious games that intervene in the rehabilitation therapy of children with autism, as shown in Figure 2.

### 2.6 Adaptive difficulty and engagement mechanics

The game is structured into five thematic “quest clusters” (Self-Care, Animal Bonding, Outdoor Exploration, Cooperative Tasks, Emotion Labeling).

Tiered Complexity: Each cluster contains three tiers. Tier-1 tasks are single-step (e.g., “feed the panda 200 kcal”), Tier-2 add social cues (virtual friend requests), Tier-3 demand joint attention (simultaneous actions with an NPC).

Dynamic Adjustment: A Bayesian learner monitors real-time performance (error rate, task latency) and modulates (i) visual clutter, (ii) NPC conversational turns, and (iii) required pedaling watts.

Flow Maintenance: Difficulty is increased when success > 80% over three consecutive trials and decreased when < 40%.

Intrinsic Motivation: Token economy (virtual badges convertible to print-out stickers) and avatar personalisation sustain engagement beyond novelty fade.

### 2.7 Game development process

The development of the interactive rehabilitation game was an intricate process that involved a multidisciplinary approach. The game’s design was rooted in the psychological and physiological needs of children with Autism Spectrum Disorder (ASD), aiming to provide a therapeutic experience through immersive technology. The process commenced with a thorough analysis of child psychology, followed by the application of game based intervention strategies. The game was designed to leverage the principles of flow theory to enhance user engagement and therapeutic outcomes.

The development team was composed of experts from various fields, including multimedia and film media art, imaging science, psychology, game design, and education. Collaborative efforts were essential, involving specialists in ASD therapy, educational psychologists, and technical professionals in virtual reality (VR) and motion-sensing technology.

### 2.8 Technical details and development process

The game was constructed using the Unity3D engine, which facilitated the creation of a three-dimensional (3D) interactive environment. The development process involved the following stages:

1. Conceptualization of game mechanics that would address the core symptoms of ASD, such as social interaction and communication skills.
2. Design of a virtual environment that simulated realistic animal interactions to promote engagement.
3. Integration of motion-sensing technology to allow for physical interaction with the game, promoting kin-esthetic learning.
4. Implementation of a serial communication protocol to interface with peripheral devices, such as the exercise bike.
5. Rigorous testing and iterative refinement based on feedback from ASD children and their therapists.

### 2.9 Equipment requirements

The game was designed to be compatible with a range of VR headsets, specifically the Hypereal VR HMD, to provide an immersive visual experience. Additional equipment included:

1. Positioning cameras for precise tracking of user movements.
2. An exercise bike equipped with an intelligent resistance system and magnetic generator for physical engagement.
3. A computer system with sufficient processing power to run the Unity3D engine and render the VR environment smoothly.

### 2.10 Data interaction with the exercise bike

The exercise bike was interfaced with the game through a serial port communication protocol. The bike’s electronics captured physical data such as pedaling speed, heart rate, and cycling distance. This data was packaged into a data stream and transmitted to the game software via a USB connection. The game software featured a script that continuously polled the serial port for incoming data, parsed the stream, and converted it into in game energy points. This energy was then used to power various activities within the game, creating a closed loop of physical exertion and virtual reward. The system was designed to ensure that the child’s physical activity levels influenced their progress and interactions within the game environment.

## 3 Method

The preliminary experiment (PE) and Formal experimental (FE) testing methods are taken to investigate the interactive VR-Motion serious game experience in improving social and learning disorders as well as life disorders in children with autism. For this study, two children with comparable autism severity were selected for the preliminary experiment. Following the successful outcomes of the PE-Test, an additional 20 children with analogous autism severity levels were recruited for the formal experiment.

Two low-grade children with autism are selected in the pre-experimental phase. These child patients are more cooperative and the duration of the interactive VR-Motion serious game intervention treatment was easier to obtain and easier to compare in terms of validity. The purpose is that through the use of this game experience, the social contact desires of autistic children can be stimulated and autistic children can be led to make social improvement from virtual game social contact to social contact in social life and cultivate excellent and correct social methods and living habits. The game entertainment will provide a happy social contact experience and life experience for autistic children and give them confidence and correct establishment of the social method. On this basis, a professional team and a monitoring team are formed to supervise the implementation of the experiment, correct sudden inappropriate behaviors of autistic children during the experiment and help to intervene in appropriate psychological guidance and comfort. (Respect for the self-adjustment willingness of autistic children in the whole process).

### 3.1 Pre-experimental stage

#### 3.1.1 PE-participants

Two autistic children around 10 years of age are selected for this experimental study. There is a control group ( $Y_{\mathrm{age}}$ =123.8 months, SD = 32.27 months) and an experimental group ( $Y_{\mathrm{age}}$ =119.4 months, SD = 29.41 months) with one male and one female.

#### 3.1.2 Observation index

To compare the improvement of two children’s daily social skills and negative emotions before and after using the immersive VR game: the results of the SDS (21) (Self-Rating Depression Scale) and the SAS (22) (Self-Rating Anxiety Scale) are used to compare the improvement of the two autistic children. The children’s daily living and social skills are assessed by using the Barthel Index Scoring (23) (100 scores as the full score).

#### 3.1.3 Intervention methods

##### 3.1.3.1 Pre-experimental stage

Control Group (*n* = 1, $Y_{\mathrm{age}}$ =123.8 months, SD = 32.27 months): Received regular intervention training without the interactive VR-Motion serious game. This included traditional therapies such as speech therapy, occupational therapy, and social skills training.

Experimental Group (*n* = 1, $Y_{\mathrm{age}}$ =119.4 months, SD = 29.41 months): Received the same regular intervention training as the control group, supplemented with interactive VR-Motion serious game training four times a week, 4 h each time, for 3 weeks.

Data were collected after the three-week training cycle for analysis of both groups. When the pre-experiment was conducted for three weeks and valid data were collected, the formal experimental policy was carried out simultaneously.

#### 3.1.4 Emotional effects of the subjects

Younger children with autism often react to environmental stimuli, which can lead to emotional stress when introduced to new places such as a gaming studio. It is crucial to consider these emotional responses when designing and implementing experimental interventions. In this study, we observed that subjects younger than 10 years old showed varying levels of emotional stress when first introduced to the serious game environment. To mitigate this, we included a familiarization period where the children could explore the game environment at their own pace before the formal experiment began. Additionally, the presence of familiar caregivers during the initial sessions helped reduce anxiety and emotional stress.

### 3.2 Formal experimental stage

#### 3.2.1 FE-participants

A total of 20 autistic children around 8 to 12 years old are selected for the formal experiment. ( $Y_{\mathrm{age}}$ : 98.76–150.23 months SD: 17.79–44.81 months $G_{\mathrm{Sex}\;\text{ratio}}$ : 5:4) The 20 autistic children were randomly numbered and divided into two groups by random number sampling. Every 10 children were divided into one group. The intervention method was the same as that of the pre-experiment.

#### 3.2.2 Randomization and blinding

Random allocation was performed by an independent biostatistician using the REDCap randomization module to generate computer-based block sequences. Participants were assigned sequential identification numbers according to their registration order. Each number was sealed in an opaque, tamper-evident envelope, the envelopes were thoroughly shuffled, and-under the joint supervision of the principal investigator and the intervention therapist-opened in the presence of the participant immediately before group assignment. This procedure ensured strict allocation concealment and maintained blinding integrity throughout the trial.

#### 3.2.3 Familiarization period and caregiver involvement

A structured two-phase acclimatization protocol preceded the formal intervention. Phase 1 (Exploratory; Day-7) allowed children 15 min of unstructured interaction with the VR headset in a quiet room, monitored for cybersickness via the Simulator Sickness Questionnaire. Phase 2 (Guided; Day-3) involved a 15 min scripted walkthrough led by the therapist, highlighting task rules and safety cues. Primary caregivers attended both sessions, receiving a 10 min briefing sheet detailing supportive prompting strategies and expected behavioral goals.

#### 3.2.4 Autism behavior checklist (ABC)

For the formal experiment, the same test mode of the intervention experiment as for the pre-experiment is used to provide an initial understanding of the present level of autism in the tested autistic children, in order for later comparison of the effects. (As shown in Table 1 for the comparison of the ABC scale scores of the three groups of children). At the end of the experiment, this scale was used to analyze the valid data of 19 tested children and compare the experiment’s effect.

**Table 1:** ABC scale score ratio of three groups of ASD children.

Scale score experimental groups	Control group 1	Experimental group 2	Experimental group 3	*t*	*p*
ABC scale score	64.87 ± 22.17	62.15 ± 18.77	63.63 ± 23.14	0.298	0.797

The ABC Scale score have five characteristics: sensory ability (s), motor ability (B), communication ability (R), language ability (L) and self-care ability (S), with a total of 57 items about the behavioral characteristics of autistic children.

According to the data in Table 1, the autism condition of the subjects is initially understood and then the random number grouping of the three groups is carried out for the experiment. It can be learned that there is no significant difference in the level of autism among the three groups participating in the experiment. Group 1 is the control group, which has carried out normal autism treatment without the interactive VR-Motion serious game. Group 2 and 3 are the experimental group, that have carried out the normal autism treatment while adding the VR-Motion serious game with different lengths of time (Group 2 is carried out the game four times a week, with five hours per time and a total of 16 weeks).

### 3.3 Statistical assumption checks

When assumptions were breached, Mann–Whitney U (between-group) or Wilcoxon signed-rank (within-group) tests supplanted parametric analyses. Exact two-tailed *p*-values and rank-biserial effect sizes are reported alongside 95% CIs. All analyses adhered to CONSORT 2010 and utilized SPSS v20.0, for the processing and analysis of the study’s data. [N(%)] is used to represent count data and $X^{2}$ test and Fisher exact test are used for comparison and analysis among groups. ( $\{{\bar{x}}_{\pm }\delta$ ) is used to indicate measurement data and comparison and analysis among groups are performed by using *t*-test. (When the difference has a statistical significance, *p* < 0.05.)

### 3.4 Outcome measures and evaluation strategy

Primary endpoint: Change in Social Responsiveness Scale-2 (SRS-2) total score from baseline (T0) to end-of-treatment (T1, Week 16). The SRS-2 is a validated, caregiver-rated instrument for autism-related social impairment.

Secondary endpoints:

1. Barthel Index of Activities of Daily Living (self-care subscale).
2. Self-Rating Depression Scale (SDS) and Self-Rating Anxiety Scale (SAS).
3. Heart-rate variability (root-mean-square of successive differences, RMSSD) during gameplay, sampled via Bluetooth chest-strap, as an objective marker of autonomic regulation.
4. Igroup Presence Questionnaire (IPQ) to quantify immersion and engagement in the VR environment.

Assessment schedule: T0 = baseline; T1 = Week 16; T2 = follow-up Week 24. All psychometric testing is administered by blinded assessors who are independent of the intervention team.

Statistical analysis plan: All analyses follow an intention-to-treat approach. A linear mixed-effects model (fixed factors = group, time; random = subject ID) estimates group × time interactions. Post-hoc contrasts employ Bonferroni correction. Effect sizes are reported as Hedges’ g with 95% CIs. Missing data are multiply imputed (*m* = 20) using predictive mean matching. Bayesian parameter estimation (weakly informative priors) supplements frequentist inference to quantify evidence strength. Adverse events—including Simulator Sickness Questionnaire scores ≥ 15—are summarized descriptively.

## 4 Result

### 4.1 Preliminary experimental results

Upon examination of Table 2, it is evident that there is a notable difference in the SDS and SAS scale assessment scores between children with autism who engaged in the interactive VR-Motion serious game intervention ( $Y_{\mathrm{age}}$ =119.4 months SD = 29.41 months) and those who did not partake in the intervention. Compared with those who do not participate in the interactive VR-Motion serious game intervention. Among them, the scores of autistic children who have carried out the game intervention are significantly lower than those who have not carried out the game intervention.

**Table 2:** SDS and SAS scores of two groups before and after carrying out serious game intervention.

Experimental group type	Index	Group	*N*	Before adding serious game	After adding Serious game	Comparison of subjects before treatment		Comparison of subjects after treatment	
						*x* ^2^	*p*	x^2^	*p*
Pre-experimental	SDS	Experimental group	1	68.63 ± 4.97	59.87 ± 5.27	0.003	0.987	0.021	2.124
		Control group	1	71.05 ± 5.61	61.26 ± 6.07				
	SAS	Experimental group	1	74.57 ± 6.98	57.67 ± 5.59	0.004	0.945	0.026	2.341
		Control group	1	78.77 ± 7.01	58.54 ± 6.13				
Formal-experimental	SDS	Control group 1	10	64.74 ± 5.18	31.68 ± 6.01	0.007	1.087	0.04	4.12
		Experimental group 2	9	65.13 ± 6.55	29.67 ± 6.09				
	SAS	Control group 1	10	74.92 ± 6.07	37.11 ± 5.79	0.006	0.988	0.033	4.47
		Experimental group 2	9	75.13 ± 6.28	34.03 ± 6.55				

### 4.2 Formal experimental results

Through three groups of experiments, the valid measured data of 19 autistic children are analyzed. It can be seen that after the use of immersive VR game to participate in the therapy, the effect has a significant difference. Among the three groups of data, experimental group 2 has the best improvement effect (using 16 weeks of VR) while the improvement effect of control group 1 (without the serious game) is significantly lower than that of the remaining groups. The data analysis of SDS and SAS scales at the end of the experiment is shown in Table 2.

The experimental data analysis shows that the design of this interactive VR-Motion serious game is relatively reasonable and it has a good effect on the development of autistic children’s social communication and self-care ability. In the post-test of wish tracking, df = 9; *t* = −1.155; *p* = 0.281, which can be seen that there is a good improvement in social intentions of autistic children after carrying out an immersive VR game experience. The experts indicate in their opinions that the design of this immersive VR game has superior guidance for simulating trial social contact and experiencing warm social emotions of children with autism spectrum disorder (ASD) and has excellent benefits in cultivating their independent willingness to participate in social contact and introducing them into life situations correctly in the game process.

The intervention treatment of experimental interactive VR-Motion serious game for autistic children through pre-experiment and formal experiment has a better improvement effect on alleviating the depression and anxiety of ASD children. By comparing the experimental SDS and SAS levels, the recovery degree of levels is better and higher after the game is intervened, which is enough to see that the positive effect in social skills and social life is substantial (see Table 2). Thus, it can prove my presupposition that this interactive VR-Motion serious game can play a positive role in the application of treating autistic children.

Since the causes of ASD and the influence factors are the combined action of multiple factors, this creates a lot of inconveniences and difficulties in the treatment of autistic children. After taking two experiments, preliminary phase conclusions have been drawn through the analysis of the data collected in the intervention treatment acting on autistic children and the results obtained from callback effect analysis of a part of selected autistic children in the experimental group are also very optimistic, as shown in Table 3.

**Table 3:** Assessment table of post-test social life ability.

Group	*N*	Before treatment	After treatment
Experimental group	9	44.09 ± 7.26	87.35 ± 8.43
Control group	10	40.87 ± 7.99	70.89 ± 7.56
*p*		0.865	3.971
*x* ^2^		0.037	0.043

**Figure 3:**
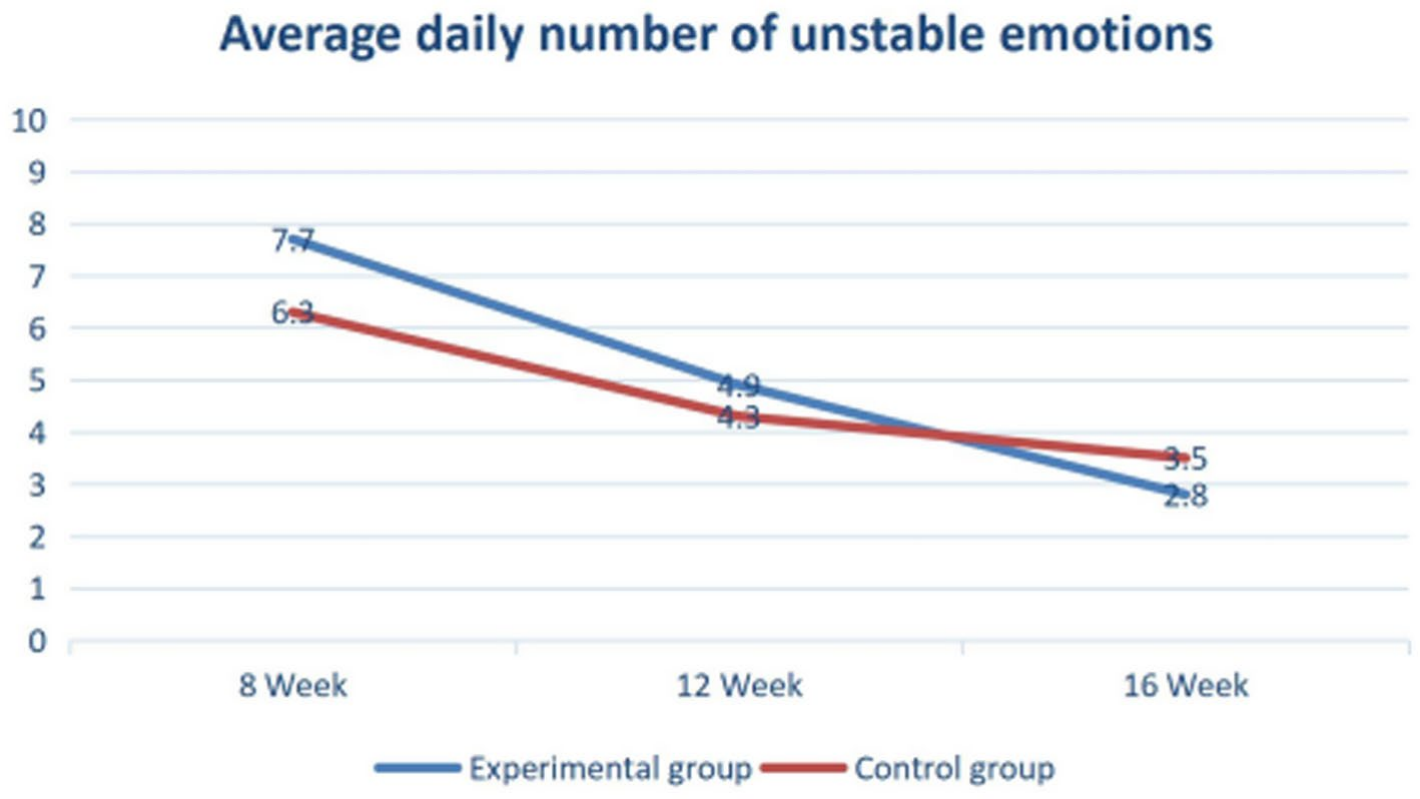
Comparison and report table of emotional stability level of ASD children.

It can be clearly seen from the Figure 3 that under the interactive VR-Motion serious game, the unstable emotional level of autistic children is gradually decreasing overall. The average unstable emotional level of the autistic children in the experimental group improved better after 14 weeks than the control group(traditional treatment) who do not receive the serious game therapy. Thus, it can be seen that this game is designed to have a significant impact on practical applications for autistic children and can be added to or used in cooperation with regular psychotherapy for autistic children, which has an excellent effect on improving their social contact willingness and life skills and stabilizing their emotions.

## 5 Discussion

The results of this study indicate that the interactive VR-Motion serious game has a positive impact on the social communication and self-care abilities of children with autism. The data analysis shows that the design of this interactive VR-Motion serious game is relatively reasonable and has a significant effect on the improvement of these skills.

### 5.1 Emotional effects

Younger children with autism often react to environmental stimuli, leading to emotional stress when introduced to new environments such as a gaming studio. During our study, subjects under the age of 10 exhibited varying levels of emotional stress when first exposed to the serious game environment. To mitigate this, we included a familiarization period where the children could explore the game environment at their own pace before the formal intervention began. Additionally, familiar caregivers were present during initial sessions to help reduce anxiety and emotional stress. The enforcement of the experiment for subjects under the age of 10 required special attention. Extra care was taken to ensure that these younger children were comfortable and engaged. For instance, breaks were provided more frequently, and the duration of gaming sessions was adjusted based on the child’s comfort level and attention span. This approach helped maintain a supportive and non-threatening environment, which is essential for the well-being and effective participation of younger subjects.

### 5.2 Drawbacks of the proposed game therapy

While the interactive VR-Motion serious game showed promising results, there are several drawbacks that need to be addressed for future improvements:

1. Limited Generalization: Although the game helped improve social skills and self-care abilities within the game environment, there was limited evidence of these skills transferring to real-world situations. Future iterations of the game should focus on enhancing the generalization of learned skills to everyday life.
2. Technological Barriers: The need for specialized equipment such as VR headsets and motion sensors can limit the accessibility of this therapy for many families. Developing more accessible and affordable versions of the game is crucial for wider adoption.
3. Emotional Stress: As mentioned, the introduction of a new gaming environment can cause emotional stress, particularly in younger children. Future designs should incorporate strategies to minimize this stress, such as gradual introduction to the game environment and involving familiar caregivers in the initial sessions.
4. Resource Intensity: The requirement for a third-party teacher to assist with the game limits the scalability of this intervention. Exploring ways to make the game more autonomous or to train parents and caregivers to facilitate the game could help address this issue.

### 5.3 Research limitations

This study has several constraints. First, the underpowered sample (N < 20) increases susceptibility to Type II error and restricts subgroup analyses. Second, the trial was conducted in a single metropolitan centre, limiting ecological and cultural generalisability. Third, intervention fidelity depended on bespoke VR hardware (~US$ 600 per unit), which may impede scalability in low-resource settings. Fourth, self-report instruments (SDS/SAS) are vulnerable to social-desirability bias, although we mitigated this by blinded administration. Finally, the absence of a long-term follow-up (> 6 months) precludes definitive conclusions on sustained efficacy. Future multisite trials with cost-effectiveness analyses are warranted.

## 6 Conclusion

This study shows that the interactive VR-Motion serious game improves the social communication and self-care skills of children with autism. The game integrates physical activity through an exercise bike, effectively engaging children in therapeutic activities. Data from both preliminary and formal experiments indicate substantial improvements in social skills and emotional stability among participating children. The experimental groups outperformed the control group, highlighting the game’s effectiveness in fostering social interaction and reducing anxiety and depression. The combination of physical exercise and interactive gameplay not only engaged children but also provided measurable emotional benefits. The game’s design, with immersive VR environments and real-time feedback from virtual animals, successfully maintained the children’s interest and engagement. Teacher involvement further enhanced the therapeutic experience.

However, challenges include the need for specialized equipment, which may limit accessibility, and ensuring skills transfer to real-world situations. Managing emotional stress caused by the new gaming environment also requires attention. Future improvements should focus on skill generalization, developing more accessible game versions, and strategies to minimize emotional stress. In summary, the interactive VR-Motion serious game is a promising tool for autism therapy, combining physical activity and interactive gameplay to support social and emotional development.

## Data Availability

The datasets presented in this article are not readily available because before the project experiment, a data confidentiality agreement had been signed with the guardian. Requests to access the datasets should be directed to 24193@tongji.edu.cn.

## References

[1] Hampton LH Harty M Fuller EA Kaiser AP. Enhanced milieu teaching for children with autism spectrum disorder in South Africa. Int J Speech Lang Pathol. (2019) 6:635–45. doi: 10.1080/17549507.2018.1559357 PMC7373342 30724622

[2] Chen L Belter M Liu J Lukosch H. Immersive virtual reality enabled interventions for autism spectrum disorder: a systematic review and meta-analysis. Electronics. (2023) 12. doi: 10.3390/electronics12112497

[3] Zhao J Zhang X Lu Y Wu X Zhou F Yang S-B. Virtual reality technology enhances the cognitive and social communication of children with autism spectrum disorder. Front Public Health. (2022) 10. doi: 10.3389/fpubh.2022.1029392, PMID: 36276341 PMC9582941

[4] Frolli A Savarese G Di Carmine F Bosco A Saviano E Rega A. Children on the autism spectrum and the use of virtual reality for supporting social skills. Children. (2022) 9. doi: 10.3390/children9020181 35204903 PMC8870236

[5] Dan Y. Potential application of virtual reality in ASD intervention. Highlights in science, engineering and technology (2023) 46:162–175. doi: 10.54097/hset.v46i.7698

[6] Wang X Young GW Guckin CM Smolic A. A systematic review of virtual reality interventions for children with social skills deficits In: 2021 IEEE international conference on engineering, Technology & Education (TALE) (2021). 436–43. doi: 10.1109/TALE52509.2021.9678808

[7] Lee I. Kinect-for-windows with augmented reality in an interactive roleplay system for children with an autism spectrum disorder. Interact Learn Environ. (2020) 29:688–704. doi: 10.1080/10494820.2019.1710851

[8] Lin J Li J She Y Lin L Wu H Zhang E. Using a social robot for children with autism: a therapist-robot interactive model. Comput Anim Virtual Worlds. (2022) 33. doi: 10.1002/cav.2109

[9] Georgiadi M Plexousakis S Vaiouli P Lithoxopoulou M. The use of robotics in enhancing social skills in school and therapeutic settings in children and adolescents with autism Spectrum disorder In: Designing, constructing and programming robots for learning (2022). 160–78. doi: 10.4018/978-1-7998-7443-0.ch008

[10] Luz V d A Going LC Toschi-Dias E. Serious games as instruments for the social habilitation of children with autism Spectrum disorder: a narrative review. Caderno Pedagógico. (2024) 21. doi: 10.54033/cadpedv21n12-039

[11] Garzotto F Gelsomini M Oliveto L Valoriani M. Motion-based touchless interaction for ASD children: a case study In: Proceedings of the 2014 international working conference on advanced visual interfaces: ACM, Association for Computing Machinery (2014). 117–20. doi: 10.1145/2598153.2598197

[12] Zhou Y Song F. Design of a cognitive training app for children with autism based on applied behavior analysis. Packaging Engineering. (2018) 39:132–9. doi: 10.19554/j.cnki.1001-3563.2018.08.027

[13] Sha Q Chen D Yu X Zhao W Le L. Design and development of virtual reality games for children with autism. China Educational Technology Equipment. (2018) 428:37–40. doi: 10.3969/j.issn.1671-489X.2018.02.037

[14] Azadboni TT Nasiri S Khenarinezhad S Sadoughi F. Effectiveness of serious games in social skills training to autistic individuals: a systematic review. Neurosci Biobehav Rev. (2024) 161. doi: 10.1016/j.neubiorev.2024.105634 38494122

[15] Ma B Li F Zeng X. A review of multimodal interaction in gaze studies: a new direction in autism research. Journal of Lanzhou University (Social Sciences). (2022) 50:112–21. doi: 10.13885/j.issn.1000-2804.2022.02.010

[16] Sha P Zhang H Liu Q. The impact of sandplay game therapy on social interaction behavior development in children with autism spectrum disorders. Chin J Spec Educ. (2022) 266:51–9. doi: 10.3969/j.issn.1007-3728.2022.08.006

[17] Chang Y Shih W Landa RJ Kaiser AP Kasari C. Symbolic play in school-aged minimally verbal children with autism spectrum disorder. J Autism Dev Disord. (2018) 48:1436–45. doi: 10.1007/s10803-017-3388-6, PMID: 29170936

[18] Schreider Sheila da Luz Souza Josiany Carlos de Freitas Éberte Valter da Silva Panceri João Antonio Campos Caldeira Eliete Maria de Oliveira Bastos-Filho T. Psychomotor intervention through serious games in children and adolescents with autism spectrum disorder using a therapeutic robot. Research Biomedical Engineering (2024) 40:485–97. doi: 10.1007/s42600-024-00358-3

[19] Shen Z Olshan SM. Efficay of sports interventions for children with autism spectrum disorder and promising targets. J Student Research. (2023) 12. doi: 10.47611/jsrhs.v12i1.3894

[20] Raaja Shri A Kamalee S Rahul B Menaka SR. Fine motor therapy using avatar-based AI for autistic children (virtual reality). International Acad J Science Engineering. (2022) 9. doi: 10.9756/IAJSE/V9I2/IAJSE0908

[21] Zung WWK. A self-rating depression scale. Arch Gen Psychiatry. (1965) 12:63–70. doi: 10.1001/archpsyc.1965.01720310065008 14221692

[22] Zung WWK. A rating instrument for anxiety disorders. Psychosomatics. (1971) 12:371–9. doi: 10.1016/S0033-3182(71)71479-0, PMID: 5172928

[23] Ahmed S Waseem H Sadaf A Ashiq R Basit H Rose S. Daily living tasks affected by sensory and motor problems in children with autism aged 5-12 years. J Health Med Nurs. (2021) 92:7–12. doi: 10.7176/JHMN/92-02

